# Rituximab as First-Line Therapy in Severe Lupus Erythematosus with Neuropsychiatric and Renal Involvement: A Case-Report and Review of the Literature

**DOI:** 10.4172/2165-7920.10001033

**Published:** 2017-10-27

**Authors:** Andrea Angeletti, Olga Baraldi, Anna Laura Chiocchini, Giorgia Comai, Paolo Cravedi, Gaetano La Manna

**Affiliations:** 1 Department of Experimental Diagnostic and Specialty Medicine (DIMES), Nephrology, Dialysis and Renal Transplant Unit, St Orsola Hospital, University of Bologna, Bologna, Italy; 2 Department of Medicine, Division of Nephrology, Icahn School of Medicine at Mount Sinai, USA

**Keywords:** Systemic lupus, Immunosuppression, First-line therapy, B cell

## Abstract

Neuropsychiatric and renal involvement are common in systemic lupus erythematosus with negative impact on patient survival. Glucocorticoids, antiproliferative and cytotoxic agents represent first-line therapies, but are often ineffective and are burdened by significant toxicities. Despite the negative results of two randomized controlled trials, rituximab is still widely used as second- or third-line therapy in similar cases. No case has been reported so far where rituximab has been used as first-line therapy.

We report the case of a 60-year-old cCaucasian woman with concurrent neuropsychiatric and renal lupus erythematous treated with one cycle of rituximab therapy at disease onset. Treatment was well tolerated and at 24 months the patient is in complete remission and free of immunosuppression. To the best of our knowledge, this is the first case of neuropsychiatric and renal lupus erythematosus successfully treated with rituximab as first-line therapy.

## Introduction

Systemic lupus erythematosus (SLE) is a chronic autoimmune disease characterized by multisystem involvement with a large spectrum of clinical manifestations. The kidney is involved in over 50% of the cases and significantly worsens the prognosis of affected patients. Less common are neuropsychiatric signs in lupus, that include heterogeneous abnormalities involving the central, peripheral and autonomic nervous system.

Therapy of lupus is a major challenge, especially for patients with renal and neuropsychiatric involvement. Steroids and antiproliferative agents represent first-line therapies, but over 35% of the patients do not respond or relapse after initial remission [1]. The crucial pathogenic role of B cells in the pathogenesis of the disease led to the hypothesis that B cell depletion could ameliorate disease natural history [2]. The two-large randomized controlled trials in extra-renal lupus (EXPLORER study) and lupus nephritis (LUNAR study) failed to achieve their primary endpoints [3,4]. Nonetheless, the excellent safety/efficacy profile of rituximab in uncontrolled series still fuels its use in the clinical practice.

The total number of circulating B cells has not been considered an element to drive B cell depleting therapy in lupus. However, it is possible to speculate that patients with higher B cell numbers are the ones where B cells play a dominant pathogenic role [2] and may mostly benefit of B cell depletion.

Herein, we report the 24-months outcome of a woman with concurrent neuropsychiatric systemic lupus erythematosus (NPSLE) and lupus nephritic (LN) who presented with high peripheral B cell counts and was successfully treated with rituximab as first-line therapy. We also provide a review of literature on rituximab in NPSLE.

## Case Presentation and Therapy

In February 2015, a 60-year-old Caucasian woman was admitted at the emergency department of St. Orsola Hospital, Bologna, Italy for generalized articular pain, fever, paresthesia of lower limbs and feet soles, resulting into unstable gear. At admission, the patient reported recent worsening of such symptoms and the appearance of visual disorders characterized by impaired ability to focus and binocular vision, despite an unspecific antibiotic therapy (amoxicillin/clavulanic acid). Clinical history was characterized by acute rheumatic pain about 40 years prior. Physical exam showed depressed mood, moderate peripheral edema, pulmonary murmur extensively reduced and basilar wet sounds, rhythmic heart sounds, no skin lesions and normochromic urine. Neurological examination was positive for internuclear left ophthalmoplegia with diplopia secondary, right facial-brachial plexus deficit, hypopallesthesia of inferior limbs and superficial weakness of the feet. Blood pressure was 125/80 mmHg, heart rate 76 bpm, body temperature 36°C, body weight of 66 kg, slightly increased over the one recorded one week prior (63 kg), urine output was 60 ml/h.

Laboratory tests showed nephritic syndrome (proteinuria 1.4 g/day and red blood cell casts in urinary sediment) with normal renal function (serum creatinine: 0.5 mg/dl; estimated glomerular filtration rate by MDRD formula: 115.6 mL/min/1.73 m^2^ [4]); The patient had positive antinuclear (1:640), anti–double-stranded DNA (anti-dsDNA) (1:1280), anti-Ro/SSA and antiphospholipid antibodies were positive. Serum complement component C3 was slight reduced and CD19+ cell count represented 38.4% of total lymphocytes (normal range: 7% to 14%). Lupus anticoagulant antibodies were positive. Anti-neutrophil cytoplasmic antibody was in the normal range. No major serum markers of systemic infection were present (white blood cells 7,990/mm^3^, C-reactive Protein 1 mg/dl). The cerebrospinal fluid was negative for autoantibodies or signs of liquor infection. Magnetic resonance imaging (MRI) reported diffuse inflammatory lesions at different developmental stages. Electroencephalogram (EEG) was compatible with minimal structural alterations. Renal ultrasound was unremarkable.

Prednisone was started at the dose of 1 g/day for 3 days. On day 4, proteinuria increased to 2 g/day and the patient was admitted in the Nephrology Unit of the St. Orsola Hospital in Bologna, Italy. Due to increased pain at left leg, we performed doppler-ultrasound that revealed deep vein thrombosis. We started sodium enoxheparin treatment (6,000 IU twice a day) that prevented performance of a renal biopsy. Overall, the pattern was consistent with SLE with simultaneously manifestations of LN and NPSLE.

In light of the high percentages of circulating B cells (Table 1) rituximab was presented to the patient as a therapeutic option and she provided her written informed consent to the treatment. The patient received 4 weekly infusions of rituximab (375 g/m^2^) after premedication with chlorphenamine (10 mg), hydrocortisone (500 mg) and paracetamol (1 g).

## Outcome

**Figure 1:**
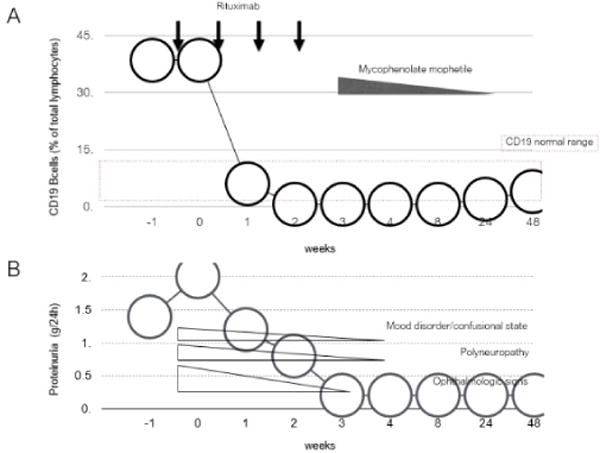
Clinical course of the patient since admission in the nephrology unit (week 0). Therapy and changes in CD19^+^ circulating B cells (A), 24 h proteinuria levels and neurological signs (B) before and up to 24 months after the first rituximab administration.

Rituximab therapy was well tolerated. Circulating CD19+ B cells were fully depleted since after the first 2 rituximab administrations and recovered to normal levels at 24 months after rituximab infusion (Figure 1).

Complete renal remission (defined as a decrease in proteinuria to less than 0.5 g/day of protein and disappearance of hematuria) was obtained at 3 weeks after starting rituximab therapy and maintained throughout the 2-year follow-up period (Figure 1 and Table 1). Neuropsychiatric disorders largely resolved at 4 weeks after starting rituximab treatment. At 1 month from the beginning of anticoagulant therapy, heparin was interrupted, and kidney biopsy was performed. The 10 available glomeruli showed glomerulonephritis with deposition of immune complexes with rare sub epithelial deposits. Immunofluorescence analysis were compatible with LN in resolution (differential diagnosis class II and class V Classification of the ISN/RPS 2004).

At 2 weeks after the last rituximab infusion, patient was treated with mycophenolate mofetil (MMF) (2 g/day), after one year it was tapered to 1 g/day and discontinued at 18 months. At 24 months after rituximab therapy, patient is in well conditions and off-treatment with no sign of disease.

## Review of the Literature

Table 2 summarizes previous reports on the efficacy of rituximab in NPSLE [6–15] (Table 2). Only Ye et al. [16] reported the use of rituximab in 6 patients with recent-onset myelitis, the others reported the use of rituximab in refractory cases of NPSLE (Table 2). Overall, data show that second-line therapy with rituximab induced remission in 33% to 100% of patients with severe refractory NPSLE [17], but randomized studies are required to define the possible role of rituximab in the therapeutic management of NPSLE. To the best of our knowledge, rituximab has never been reported as first-line therapy in patients with NPSLE and lupus nephritis.

## Discussion

Herein, we reported the case of an adult patient with concurrent presentation of LN and NPSLE who achieved complete and sustained neurological and renal remission after receipt of rituximab as first-line therapy. Treatment was well-tolerated and allowed lowering of concomitant immunosuppression with methylprednisolone.

In contrast to other systemic manifestations of SLE, the prominent feature of NPSLE is not the presence of vasculitis [18]. Instead, growing evidence shows that blood–brain barrier (BBB) dysfunction is essential to the development of NPSLE, allowing the passive diffusion of auto-reactive antibodies and cytokines, facilitating the development of a pro-inflammatory features [19,20].

There are no high-quality studies testing treatment for NPSLE and, similarly to LN, no specific guidelines have been published [21]. Evidence indicates that B cells play a crucial role in the pathogenesis of the lupus, both in renal and neurological manifestations, which supports B cell-depletion [22] to selectively treat disease without the devastating consequences of unspecific immunosuppression [23], also and in previous reports where rituximab was used as second-line therapy (Table 2).

In our case, the decision to utilize rituximab as first-line therapy, rather than cyclophosphamide [24], was mainly driven by the high number of circulating B cells and, as previously described [19,20], by the specific features of NPSLE compared to other systemic manifestations of SLE. Intriguingly, after full B-cells depletion induced by rituximab, the B cells recovered only partially and were steadily within the normal range over the 2-year follow-up, suggesting a relationship between B cell number and disease activity. Long-term treatment with MMF may have also contributed to the persistence of remission, but it is unlikely responsible for the fast-clinical remission observed.

## Conclusion

In conclusion, we report a case of SLE with concurrent renal and nervous system involvement treated with rituximab at disease onset that developed complete remission with no side effects at 24 months of follow-up. Therefore, we believe, that rituximab could be considered in similar cases, especially if B cell count is high and use of other immunosuppressive agents is contraindicated.

## Figures and Tables

**Table 1:** Biological parameters before (month 0), and at 1 and 24 months after first rituximab administration.

Biochemical tests	Months after rituximab treatment		
	0	1	24
Serum Creatinine (mg/dl)	0.5	0.5	0.5
Proteinuria (g/24 h)	2	<0.2	<0.2
Hematuria (RBC/uL)	200	0	0
Hemoglobin (g/dl)	11	10.5	11.6
Serum albumin (g/dl)	2.3	−	3.4
Platelets (n/mm^3^)	134,000	138,000	143,000
Complement C3 (mg/dl)	84	85	96
CD19^+^ cell count (% of lymphocytes)	36.4	0.1	9.2
Anti-DNA Ab (titer)	1:1280	1:320	1:320
NPSLE features*			
Mood disorder	+++	+	−
Confusional state	+++	−	−
Polineuropathy	++++	−	−
Ophthalmologic signs	+++++	−	−
MRI	Inflammatory lesions	−	Negative

* Score Range: − Negative, + Minimum, +++++ Maximum.

**Table 2:** Major published clinical studies testing rituximab in NPSLE.

Reference	N	F/U (mo)	Study design	Previous therapy	Renal Involvement	Dose of rituximab	CR (%)	Comments
Iaccarino et al. [6]	9	12	ROS	Steroids, CYC, MMF/AZA	n.d.	375 mg/m^2^ (x4–6) or 1 g (x2)	55	Data from a multicentre registry in patients with SLE refractory to standard therapy
Hickman et al. [7]	6	6–12	Case series	Steroids, CYC, MMF/AZA, MTX	2	1 g (x1–2)	33	Rituximab was associated with significant clinical benefits in refractory NPSLE
Dale et al. [8]	18	18	Case series	Steroids (17), IVIg (9), CYC (9), PE (4), MMF/AZA (4), Hydro (4)	none	375 mg/m^2^ (x4)	40	Authors divided rituximab effect into definite (n=5), probable (n=7), possible (n=5) and no effects (n=1)
Braun-Moscovici et al. [9]	1	12	Case report	Steroids, CYC	1	375 mg/m^2^ (x4)	100	Complete remission with only a residual left foot drop
Sanz et al. [10]	1	N/A	Case report	Steroids, PE, IVIg, CYC, CNI	none	500 mg (x4)	100	Patient had nephrotic syndrome: after rituximab proteinuria decreased from 6g/24h to 2.9 g/24.
Fernandez-Nebro et al. [11]	27	20 (5–35)	ROS	Steroids, Hydro, CYC, AZA/MMF, MTX	n.d.	375 mg/m^2^ (x4–6) or 1 g (x2)	75	LESIMAB study showed rituximab as an effective treatment option for patients with refractory SLE
Vital et al. [12]	13	6	ROS	Steroids, CYC et al. n.d.	none	1 g (x2)	92	Incomplete B cell depletion at 6 weeks was associated with lower clinical response rates at 6 months
Pinto et al. [13]	12	12	ROS	Steroids, Cyclo, MMF/AZA, MTX, CNI	n.d.	1 g (x2)	75	Before rituximab therapy, all patients were in treatment with two o more immunosuppressive agents
Narvåez et al. [14]	35	9 (4–33)	ROS	Steroids (33), Hydro (8), CYC (28), AZA/MMF (18), PE (10), MTX (5), IVIg (4), VCR (1)	12	375 mg/m^2^ (x4–6) or 1 g (x2)	50	45% of patients with partial or complete remission relapsed after a median of 17 months despite maintenance therapy
Tokunaga et al. [15]	10	1–12	Case series	Steroids (10), CYC (8), CNI (3), PE (4), MMF/AZA (4), MTX (4), VCR (1)	none	375 mg/m^2^ (x4–6) or 1 g (x2)	100	At flow cytometric analysis rituximab depleted both B and T cells
Ye at al. [16]	6	22 (14–37)	Case series	none	none	500 mg (x2–3)	67	Recent onset severe lupus myelopathy

* Data are presented as common follow-up period for all the patients or as median (range); N: Number of Patients; CR: Complete Remission; ROS: Retrospective Observational Study; SLE: Systemic Lupus Erythematosus; NPSLE: Neuropsychiatric Systemic Lupus Erythematosus; CYC: Cyclophosphamide; MMF: Mycophenolate Mofetil; AZA: Azathioprine; MTX: Methotrexate IVIg: Intravenous Immunoglobulin; PE: Plasma Exchange; CNI: Calcineurin Inhibitor; VCR: Vincristine; Hydro: Hydroxychloroquine.
